# Social connection profiles of older adults as both heritable and modifiable influences on brain health: data from the Older Australian Twins Study

**DOI:** 10.1002/alz.091133

**Published:** 2025-01-09

**Authors:** Suraj Samtani, Anne‐Nicole Casey, Anbupalam Thalamuthu, Ben C.P. Lam, Vibeke S. Catts, Henry Brodaty, Perminder S. Sachdev

**Affiliations:** ^1^ Centre for Healthy Brain Ageing (CHeBA), University of New South Wales, UNSW Sydney, NSW Australia; ^2^ Centre for Healthy Brain Ageing (CHeBA), UNSW Sydney, Sydney, NSW Australia; ^3^ La Trobe University, Melbourne, VIC Australia

## Abstract

**Background:**

Social connections are associated with brain health, but the extent to which social connections are heritable remains unclear. Using longitudinal twin data, we explored the heritable and environmental contributions to social connections. We hypothesised that social connections patterns would be moderately heritable and would be associated with better cognitive and mental health over time.

**Method:**

We analysed data from 333 monozygotic and 266 dizygotic twins aged 65+, collected at baseline, 2‐ and 4‐year follow‐up in the Older Australian Twins Study. Outcome variables were depressive and anxious symptoms, MMSE and neuropsychological subtest scores, and social activity items. We ran exploratory structural equation modelling, univariate heritability models, genetic correlations and linear mixed‐effects, and Inference about causation from examination of familial confounding (ICE FALCON) modelling. Fully adjusted models controlled for age, sex, education, hypertension, diabetes, smoking, alcohol use, history of depression, BMI, exercise, and additionally for APOE4 status and hearing loss (for cognition) and pain and neuroticism (for mental health).

**Result:**

We identified three social connection factors with weak to no heritability: interacting with friends and community (h^2^ = 0.09,95%CI: 0.00,0.44), family interactions and childcare (h^2^ = 0.13,95%CI: 0.00,0.43); and involvement in religious groups and caregiving (h^2^ = 0.00,95%CI: 0.00,0.19). Genetic correlations indicated overlap in genetic influences for friend interactions with global cognition (*r* = 0.21), depressive (*r* = ‐0.96), and anxious symptoms (*r* = ‐0.47). Shared environmental correlations between friend interactions and global cognition (*r* = 0.24), depressive (*r* = ‐0.53), and anxious symptoms (*r* = ‐0.54) indicated some overlap in environmental influences. Friend interactions were associated with fewer depressive symptoms cross‐sectionally (*B* = ‐0.14, adjusted *p* = 0.004) and longitudinally (*B* = ‐0.09, adjusted *p*‐value = 0.010); social connections were not associated with cognition. ICE FALCON analyses did not reveal causal relationships between social factors and cognitive/mental health.

**Conclusion:**

Certain social connection profiles were weakly heritable (friend and family interactions) and were associated with brain health (friend interactions). There was moderate overlap in the genetic and shared environmental influences affecting social connections and cognitive and mental health. Interacting with friends was associated with fewer depressive symptoms; social connections were not associated with cognitive change over 4 years. Health care for older adults should include paying attention to social connections to support brain and mental health.

